# Frameshifting in the P6 cDNA Phage Display System

**DOI:** 10.3390/molecules15129380

**Published:** 2010-12-20

**Authors:** Cindy Govarts, Klaartje Somers, Piet Stinissen, Veerle Somers

**Affiliations:** Biomedical Research Institute, School of Life Sciences, Transnationale Universiteit Limburg and Hasselt University, Diepenbeek, Belgium

**Keywords:** cDNA phage display, *E. coli*, frameshifting, p6, filamentous phage

## Abstract

Phage display is a powerful technique that enables easy identification of targets for any type of ligand. Targets are displayed at the phage surface as a fusion protein to one of the phage coat proteins. By means of a repeated process of affinity selection on a ligand, specific enrichment of displayed targets will occur. In our studies using C-terminal display of cDNA fragments to phage coat protein p6, we noticed the occasional enrichment of targets that do not contain an open reading frame. This event has previously been described in other phage display studies using N-terminal display of targets to phage coat proteins and was due to uncommon translational events like frameshifting. The aim of this study was to examine if C-terminal display of targets to p6 is also subjected to frameshifting. To this end, an enriched target not containing an open reading frame was selected and an E-tag was coupled at the C-terminus in order to measure target display at the surface of the phage. The tagged construct was subsequently expressed in 3 different reading frames and display of both target and E-tag measured to detect the occurrence of frameshifting. As a result, we were able to demonstrate display of the target both in the 0 and in the +1 reading frame indicating that frameshifting can also take place when C-terminal fusion to minor coat protein p6 is applied.

## 1. Introduction

Phage display is a high-throughput molecular technique that has been used successfully to select targets for any given ligand. Targets can be easily displayed on the surface of the phage virion by coupling the foreign DNA to a gene encoding a phage coat protein. After infection of the host, phage protein components are produced by the protein translation machinery of the infected bacterial cell and the foreign DNA will be displayed as a fusion product to one of the phage coat proteins [1]. Because of the physical link between genotype and phenotype, filamentous phage displaying a relevant polypeptide will be retained during affinity selections on candidate binding ligands followed by identification of the target [2].

**Figure 1:**
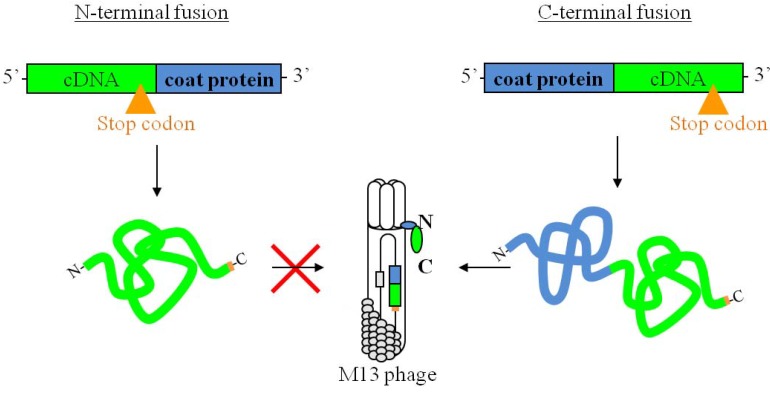
N- and C-terminal fusion of cDNA to a phage coat protein.

The most commonly used phage coat proteins for fusion are minor coat protein 3 (p3) and major coat protein 8 (p8) [3,4]. When using these coat proteins, N-terminal fusion of the target is mandatory for successful phage propagation. For the display of cDNA libraries, N-terminal fusion is not possible due to inherent stop codons present in the cDNA fragments, as represented in Figure 1. However, the free carboxyl terminus of minor coat protein 6 (p6) allows successful fusion of the cDNA without interfering with phage propagation [5]. Using the pSP6 phagemid vector which was specifically designed to enable C-terminal fusion of targets to p6, we and others have already successfully identified a variety of targets [5,6,7,8,9,10,11].

In our previous studies using C-terminal fusion of cDNA to p6, we observed that during affinity selections a small percentage of phages containing a stop codon immediately after gene VI (gVI) became enriched. This stop codon resulted from an out of frame insertion of the cDNA into the phagemid vector thereby preventing the display of the corresponding protein. Although we initially thought that the isolation of these clones could be the result of aspecific binding, the enrichment of identical cDNA sequences containing this stop codon suggested specific interactions of a displayed target with a ligand. This display could occur when unusual translational recoding such as frameshifting or ribosome hops take place. 

Frameshifting is well established within the field of phage display and bacterial expressionsystems [12,13,14,15,16]. Because previous studies were based on N-terminal fusion to p3 or p8, frameshifting had to occur in order to express the p3 and p8 coat proteins that enable phage propagation. However, C-terminal fusion to p6 does not require expression of the inserted cDNA for successful phage propagation as represented in Figure 1 [5,6]. Therefore, other methods must be used to detect the possible occurrence of these unusual translational events.

**Figure 2:**
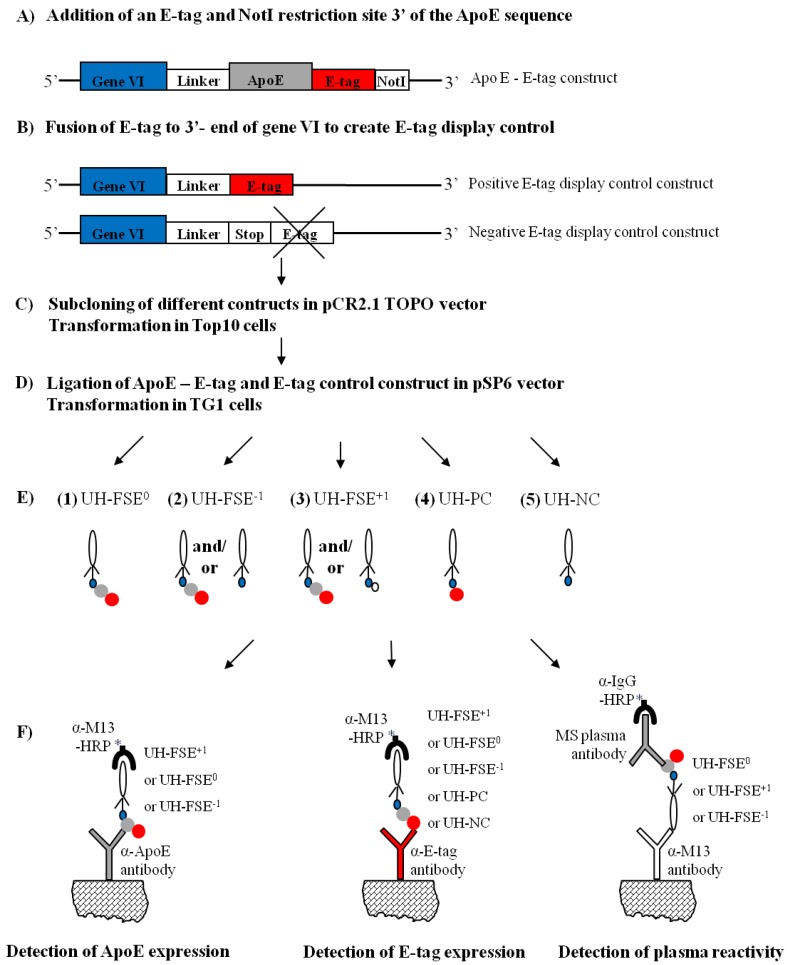
Overview of the study. First the E-tag (red) was coupled to the 3’ site of the ApoE sequence (grey) (**A**) and E-tag display controls were made (**B**). After cloning the constructs into the pCR2.1 TOPO vector and pSP6 vectors (**C-D**), different phages were purified (**E**). Phage UH-FSE^0 ^correctly displays the ApoE polypeptide (grey) and E-tag (red) fused to p6 (blue) (**E1**). UH-FSE^−1^ (**E2**) and UH-FSE^+1 ^(**E3**) express the ApoE construct with, respectively, a −1 and +1 shift in the reading frame. Although these phages normally do not express the ApoE polypeptide, part of them will if frameshifting would occur. A positive (**E4**) and negative (**E5**) E-tag display control was also purified. Expression of both ApoE polypeptide and E-tag were subsequently measured by ELISA (**F**).

In the work described here, a phage clone UH-FS was chosen for further study of frameshifting in the p6 display system. UH-FS was previously isolated after affinity selecting a multiple sclerosis (MS) cDNA display library against antibodies present in MS sera [10]. Although the inserted cDNA sequence of the phage clone encodes part of the Apolipoprotein E protein (ApoE), out of frame insertion of the cDNA sequence into the pSP6 phagemid vector resulted in an early stop codon thereby preventing the display of the protein. Fusion of an E-tag to the ApoE cDNA and subcloning of the fragment in 3 reading frames in the pSP6 vector resulted in the expression of the ApoE - E-tag construct in all reading frames. Measuring expression of both the ApoE polypeptide and the E-tag via ELISA revealed an increased expression both in the correct reading frame but also in the +1 reading frame, indicating the occurrence of frameshifting in the p6 display system. In addition we could demonstrate an increased antibody reactivity towards the correctly displayed ApoE polypeptide in the plasma of the MS patients used for the selection rounds, which strongly indicates the specific enrichment of UH-FS. An overview of the study is represented in Figure 2.

## 2. Results and Discussion

### 2.1. Fusion of an E-tag to the ApoE cDNA Sequence

**Figure 3:**
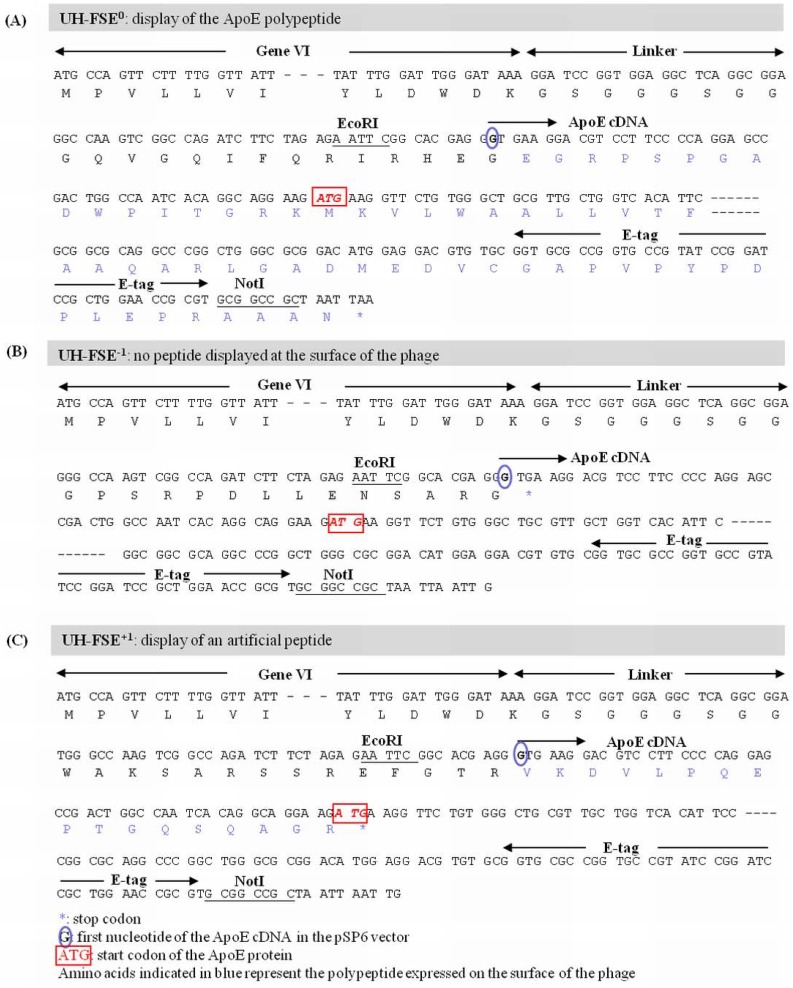
(**a**) ApoE in UH-FSE^−1^; (**b**) UH-FSE^0^ and (**c**) UH-FSE^+1^.

The inserted cDNA sequence of UH-FS encoded part of the signal sequence in addition to the first 130 amino acids (AA) of the ApoE protein. However, due to out of frame insertion of the ApoE gene in the pSP6 phagemid vector, a stop codon was observed immediately after gVI that prevented the display of the corresponding ApoE polypeptide. In order to detect whether this clone could be subjected to frameshifting, an E-tag was cloned in frame at the 3’-end of the ApoE cDNA as depicted in Figure 2A in order to obtain simultaneous expression of the ApoE polypeptide and the E-tag. This ApoE - E-tag construct was then subcloned into the pSP6 vector in 3 different reading frames (Figure 2E). This resulted in the insertion of the ApoE - E-tag in the 0 frame (designated UH-FSE^0^, Figure 3A) which represents the correct reading frame for the ApoE polypeptide to be expressed, the −1 frame (designated UH-FSE^−1^, Figure 3B) where a stop codon prevents the display of a peptide and the +1 frame (designated UH-FSE^+1^, Figure 3C) in which an artificial peptide of 17 AA is displayed at the surface of the phage. As a control for E-tag expression, the E-tag alone was inserted in the pSP6 vector in the 0 frame (designated UH-PC) and in the −1 frame (designated UH-NC) resulting in respectively high and no E-tag expression (Figure 2B). 

### 2.2. Detection of Protein Display on the Surface of the Phage

**Figure 4:**
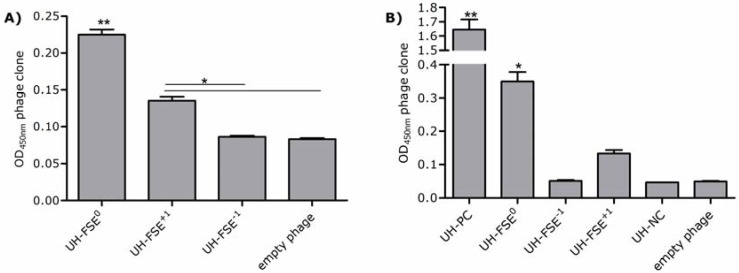
(**A**) Detection of ApoE expression. ApoE expression of UH-FSE^0^, UH-FSE^−1^ and UH-FSE^+1 ^was measured via ELISA. Empty phage was used to determine background values. Experiments were performed twice independently. All samples were tested in duplicate in one ELISA. ** *p* < 0.001; * *p* < 0.01 as evaluated by one-way ANOVA followed by Tukey post-hoc test; (**B**) Detection of E-tag expression. E-tag expression of UH-FSE^0^, UH-FSE^−1^ and UH-FSE^+1 ^was measured via ELISA. UH-PC was used as a positive E-tag display control whereas UH-NC was used as a negative E-tag display control. Empty phage was taken along to determine background values. Experiments have been performed 4 times independently, all samples were tested in duplicate. ** *p* < 0.001; * *p* < 0.01 except for UH-FSE^0^*vs.* UH-FSE^+1^*p* < 0.05 as evaluated by one-way ANOVA followed by Tukey post-hoc test.

To confirm expression of the ApoE polypeptide on the surface of the UH-FSE^0^ phage and verify possible frameshifting in the other 2 phages UH-FSE^−1^ and UH-FSE^+1^, an antibody directed against the N-terminal part of the ApoE protein was used. An empty phage, not displaying a fusion peptide, was taken along as a negative control to determine the background value. Representative data of individual experiments are shown in Figure 4a. UH-FSE^0 ^displayed a positive signal for the ApoE polypeptide confirming the display of the ApoE cDNA. Whereas UH-FSE^-1^ did not express the ApoE polypeptide, a significant increase in ApoE expression was observed for UH-FSE^+1^. These results indicate that in a fraction of the purified UH-FSE^+1^ phages, the reading frame has shifted in the −1 direction resulting in the display of the ApoE polypeptide.

### 2.3. Detection of E-tag Expression

To confirm the results of the ApoE ELISA, expression of the E-tag, fused to the ApoE sequence, was also measured as representatively depicted in Figure 4b. An empty phage was used to determine background values whereas UH-PC and UH-NC represent respectively a positive and negative control for E-tag display. A high E-tag expression was observed for both the positive control UH-PC and UH-FSE^0^ which confirmed the correct expression of the E-tag and concordant ApoE polypeptide in this reading frame. The lower E-tag expression of UH-FSE^0^ compared to the positive control can be explained by the fact that high-level expression of the ApoE polypeptide could result in some level of toxicity or growth bias to the bacterial host. This bias can be a consequence of the size (130 amino acids), the eukaryotic nature and/or possible interference of the polypeptide in the phage production process. This would in turn result in a low percentage of UH-FSE^0^ phages expressing the ApoE polypeptide and E-tag which could also explain why the UH-FSE^+1^ clone was selected during our selection procedure instead of UH-FSE^0^.

Although the significant increase in signal of the UH-FSE^+1^ clone observed in the ApoE ELISA could not be confirmed with the E-tag antibody, a higher E-tag signal was observed for the UH-FSE^+1^ clone compared to the UH-FSE^−1^ clone. E-tag expression of UH-NC and UH-FSE^−1^ again equaled background values confirming that no E-tag is displayed at the surface of these phages.

### 2.4. Specific Enrichment of Frameshifted Phage Clone UH-FS

**Figure 5:**
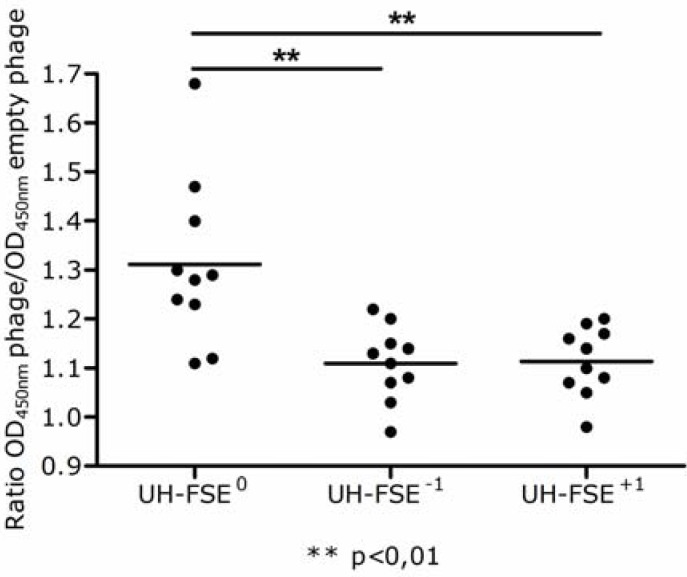
Detection of ApoE-immunoreactivity in the plasma of MS patients. Immunoreactivity towards the different UH-FSE phages was measured in the individual plasma of 10 MS patients that were used for the selection rounds. UH-FSE^0^ displays the ApoE protein in the correct reading frame. UH-FSE^−1^ and UH-FSE^+1^ represent respectively a −1 and +1 shift in the reading frame of ApoE. Background values were determined by measuring reactivity towards an empty phage not displaying any protein. Results are given as a ratio of the OD_450nm_ specific phage/OD_450nm_ empty phage.

If the original enrichment of UH-FS during the selection rounds on MS plasma would be the result of specific interactions between the frameshifted ApoE polypeptide and plasma antibodies, antibodies towards this ApoE polypeptide should be present in the plasma used for the selection rounds [10]. For that reason, an ELISA was performed where reactivity towards UH-FSE^0^, UH-FSE^−1^, UH-FSE^+1^ and empty phage was analyzed in the plasma of the 10 MS patients used for the selection rounds. Figure 5 clearly shows an increased antibody reactivity towards UH-FSE^0^ compared to UH-FSE^−1^ and UH-FSE^+1^ indicating that ApoE-specific antibodies are indeed present in the plasma of these MS patients resulting in the enrichment of UH-FS during the selection procedure.

By cloning an E-tag in frame with the ApoE cDNA, we were able to monitor the display of the cDNA at the surface of the phage. This was achieved either by an antibody directed to the polypeptide itself or directed to the E-tag fused to the polypeptide. Our results clearly indicate that for this cDNA fragment, frameshifting occurs in a proportion of the phages resulting in the expression of the ApoE polypeptide and attached E-tag in the +1 reading frame. In addition, an increased immunoreactivity towards the ApoE polypeptide could be observed in the plasma of MS patients that was used for the selection of UH-FS, which indicates that enrichment of UH-FS was the result of specific interactions between the frameshifted ApoE polypeptide displayed on the phage and plasma antibodies. 

The phenomenon of frameshifting has implications for affinity selection procedures applied on complex cDNA libraries since frameshift mutations can revert display phenotypes on phage in the presence of a suitable selection pressure. As a result, a single phage clone could encode different antigenic targets. Although this can benefit the complexity of the library, deducing the correct peptide sequence that has been selected by the selector molecule can be difficult, implying the need to verify the selection specificity in a phage-free format (*i.e.* purified peptides or recombinant proteins).

## 3. Experimental

### 3.1. Serological Antigen Selection of a Multiple Sclerosis cDNA Phage Display Library on Patient Plasma

Serological antigen selection (SAS) was applied on a MS cDNA phage display library as described previously [10]. A cDNA phage clone UH-FS (short for University Hasselt-frameshifting) was selected multiple times after affinity selecting an MS cDNA phage display library against MS plasma and was chosen for analysis of frameshifting. The cDNA of UH-FS was identical to the nucleotide sequence of the ApoE sequence (from nucleotide position −50 to 389). However, after translation of the nucleotide sequence an early stop codon was observed due to out of frame insertion of the cDNA in the pSP6 phagemid vector which prevented the display of the protein. 

### 3.2. Generation of E-tag Constructs

An overview of the study is represented in Figure 2. An ApoE - E-tag construct was generated byC-terminal fusion of an E-tag (GAPVPYPDPLEPR) and *Not*I restriction site to the ApoE sequence of UH-FS using the ApoE forward and E-tag reverse primer (see Figure 2A and Table S1 for primer sequences in the appendix). A negative control (NC) for E-tag display was generated by an out of frame fusion of the E-tag to the 3’-end of gVI (Figure 2B), resulting in a stop codon preventing the display of the E-tag. This was done using the overlapping ApoE forward and the NC reverse primer. A positive control (PC) for E-tag display was generated by using the overlapping ApoE forward and PC reverse primer. Addition of an extra nucleotide to the PC reverse primer resulted in an in frame fusion of the E-tag at the 3’-end of gVI. All primers were obtained from Eurogentec (Seraing). PCR products were purified using the GFX gel band purification kit (GE Healthcare), ligated into the pCR2.1 TOPO vector and transformed into *Escherichia coli* (*E. coli*) Top10 cells according to manufacturer’s instructions (TopoTA cloning kit, Invitrogen; Figure 2C). Insert sequences were confirmed using M13 forward and reverse primer. After *Bgl*II and *Not*I digestion, the ApoE – E-tag and the E-tag control constructs were ligated into the pSP6 vectors digested with the same restriction enzymes (Figure 2D). Ligation mixtures were used to transform *E. coli* TG1 cells by electroporation to obtain UH-FSE^0^ (correct reading frame of the ApoE gene), UH-FSE^−1^ (−1 shift in the reading frame of the ApoE gene), UH-FSE^+1^ (+1 shift in the reading frame of the ApoE gene), UH-PC (positive E-tag display control) and UH-NC (negative E-tag display control) as depicted in Figure 2D. Resulting colonies were picked and sequence was confirmed with gVI forward primer. Phage were purified as described previously [17]. Purified phage clones were used in ELISA to analyse immunoreactivity towards the displayed ApoE protein on the one hand and the level of E-tag expression on the other hand (Figure 2F).

### 3.3. Detection of ApoE and E-tag Expression by ELISA

ApoE and E-tag expression of purified phage clones UH-FSE^+1^, UH-FSE^0^, UH-FSE^−1^, UH-PC and UH-NC was measured by ELISA as shown in Figure 2F. Empty phage not displaying a peptide was taken along as a negative control to set the background value. The cutoff for a positive signal was set at 1.5 times the background value. Briefly, 96-well flat-bottomed microtiter plates (Falcon/BD) were coated overnight at 4 °C with 200 μL anti-ApoE antibody (LifeSpan BioSciences) or anti-E-tag antibody (GE Healthcare), 10 μg/mL in coating buffer (0.1 M sodium hydrogen carbonate pH 9.6) and blocked with 200 μL of 2% MPBS (2% (w/v) skimmed milk powder in PBS, 750 mM NaCl, 40 mM Na_2_HPO_4_, 7.8 mM KH_2_PO_4_, pH 7.4) for 1 h at room temperature (RT). PEG (20% polyethylene glycol - 2.5 M NaCl) - purified phage (10^10^ phage/well) were preblocked with 2% MPBS in a 96-well round-bottomed plate (Nunc) for 1h at 37 °C, followed by 30 min shaking at RT. After washing with 0.1% (v/v) PBS/Tween 20 (PBST) and PBS, the preblocked phage were transferred to the antibody-coated plate and incubated for 1h at 37 °C and 30 min shaking at RT. After washing the plate with 0.1% PBST and PBS, 150 μL of a peroxidase conjugated anti-M13 monoclonal antibody (Amersham/Pharmacia/Biotech), diluted 1:5,000 in 2% MPBS was incubated for 1h shaking at RT. After washing the plate with 0.1% PBST and PBS, 130 μL of a 3,3’,5,5’ tetramethylbenzidine dihydrochloride chromogen solution (10 mg/mL) was added. Colour development was stopped with65 μL/well 2 M H_2_SO_4_. The plates were read at 450 nm in a Bio-Rad Benchmark microplate reader(Bio-Rad). ApoE experiments were performed twice independently whereas experiments with the anti-E-tag antibody were performed four times independently. All samples were tested in duplicate in one ELISA. Statistical analysis was performed by one-way ANOVA followed by Tukey post-hoc test.

### 3.4. Measuring ApoE-antibody reactivity in plasma of MS patients

Antibody reactivity towards the ApoE polypeptide was measured in the plasma of 10 MS patients used for the selection of UH-FS. Reactivity towards UH-FSE^+1^, UH-FSE^0^, UH-FSE^−1^ was measured as described previously [7].

## 4. Conclusions

In this study we analyzed the occurrence of frameshifting in the p6 phage display system. By cloning a cDNA sequence in 3 different reading frames in a pSP6 phagemid vector, we were able to demonstrate the translation of the corresponding cDNA not only in the correct reading frame but also in the +1 reading frame. These results indicate that part of the phages was subjected to frameshifting leading to the in-frame expression of the ApoE polypeptide. Our results indicate that translational events like frameshifting also occur in the p6 phage display system emphasizing the need to confirm ligand-target binding properties in a phage-free assay using synthetic peptides or recombinant proteins.

## Appendix

**Table S1:** Primers used in the study.

**Name**	**Sequence**
ApoE forward primer	GGATCCGGTGGAGGCTCAGGCGGAGGGCCAAGTCGGCCAGATCTTCTAGAGAATTC
E-tag reverse primer	*GCGGCCGC*ACGCGGTTCCAGCGGATCCGGATACGGCACCGGCGCACCGCACACGTCCTCCAT
NC reverse primer	*GCGGCCGC*ACGCGGTTCCAGCGGATCCGGATACGGCACCGGCGCACCCCTCGTGCCGAATT
PC reverse primer	*GCGGCCGC*ACGCGGTTCCAGCGGATCCGGATACGGCACCGGCGCACCACCTCGTGCCGAATT
M13 forward primer	CAGGAAACAGCTATGAC
M13 reverse primer	GTAAAACGACGGCCAG
gVI forward primer	TTACCCTCTGACTTTGTTCAGG

All primers are listed from 5’ to 3’. Overlapping nucleotides are indicated in bold. The underlined bases represent the E-tag sequence while italic text represents *Not*I restriction site.
